# Clinicopathologic evaluation of granuloma annulare: Study of 136 Iranian cases, south of Iran

**DOI:** 10.1002/ski2.299

**Published:** 2023-10-07

**Authors:** Fatemeh Sari Aslani, Fatemeh Pouraminaee, Mozhdeh Sepaskhah, Sheida Khosravani Ardakani

**Affiliations:** ^1^ Molecular Dermatology Research Center Department of Dermatology School of Medicine Shiraz University of Medical Sciences Shiraz Iran; ^2^ Maternal‐Fetal Medicine Research Center Shiraz University of Medical Sciences Shiraz Iran; ^3^ Department of Pathology School of Medicine Shiraz University of Medical Sciences Shiraz Iran

## Abstract

**Background and Objectives:**

Granuloma annulare (GA) is a benign skin disorder with various histopathologic features that are rarely investigated in Iranian population. We performed this study to find out the clinical and histopathologic features of GA in our referral centre.

**Methods:**

One hundred‐thirty‐six patients with biopsy‐proven GA were reviewed. Clinical data and pathological features were recorded.

**Results:**

One hundred‐eight female patients and 28 male patients (Female/male ratio: 3.85) with mean age of 42.54 ± 21.2 years (range: 2–83 years) were recruited. Eighty‐eight (64.7%) patients had interstitial infiltrative pattern and 48 (35.3%) patients had complete palisading granulomas. The infiltrate occupied both upper and lower dermis in most of the cases (67.7%). Significant mucin was detected more commonly in complete GA compared to interstitial GA (*p* = 0.019), but inflammation degree, eosinophils, plasma cells, and giant cells were not different between two subtypes of GA (*p* > 0.05). The significant inflammation contained more significant plasma cells (*p* = 0.006). The significantly more giant cells were detected in patients between 20 and 60 years of age (*p* = 0.015); but other factors were not different between age groups.

**Conclusions:**

In our study, the prevalence of GA in women was significantly higher than in men. Interstitial GA was the more common histological subtype and the inflammation was less severe and the infiltrate was mostly pandermal in our cases. More severe inflammation contained more plasma cells, and more dense giant cells were seen in middle aged patients.

1

**What is already known about this topic?**

- Granuloma annulare (GA) is a benign skin disorder with various histopathologic features.
- GA is more prevalent in middle‐aged and female patients.
- Interstitial GA is the more frequent histological type of disease, and mucin is a common finding, especially in the complete type.

**What does this study add?**

- The intensity of inflammation may be less severe than the previous studies.
- More severe inflammation contains more plasma cells
- More dense giant cells could be seen in middle aged patients.

## 2 INTRODUCTION

Granuloma annulare (GA) is a self‐limited and benign disease in all age groups. It consists of grouped papules in an enlarging annular shape. It is asymptomatic, however, some cases may have mild pruritus. It might occur anywhere on the body but mostly occurs on the lateral or dorsal surfaces of the hands and feet.^1^, ^2^ Although the cause is unknown, but an association with diabetes mellitus, human immunodeficiency virus infection, malignancies (lymphoma and prostate carcinoma), and insect bites are reported.^3^, ^4^, ^5^ It is important to make a distinction between GA with other annular lesions such as tinea cruris, pityriasis rosea, sarcoidosis, Hansen's disease, urticaria, subacute cutaneous lupus erythematosus, and erythema annulare centrifugum.^6^, ^7^, ^8^ The diagnosis relies on clinicopathological correlation. The histological features of GA include collagen degeneration, palisading granulomas, mucin, and a lymphohistiocytic infiltrate.^9^, ^10^ In a study conducted by Umbert et al., the most common histological pattern of GA was infiltration of histiocytes between collagen fibres, with minimal necrobiosis (72%), followed by the palisading granulomas (25%), and the epithelioid nodules (3%). The electron microscopic findings were histiocytes with membrane activity, large mitochondria, numerous lysosomes, and well‐developed rough endoplasmic reticulum.^11^ clinical presentation of GA is classified into localized, generalized/disseminated, subcutaneous, and perforating types.^9^, ^12^

A few studies are present assessing the histological features of GA in Iranian patients; so, we conducted this retrospective, cross‐sectional study to evaluate the clinicopathological features of GA in this population.

## 3 MATERIALS AND METHODS

### 3.1 Study population

In this retrospective cross‐sectional study, we recruited all the biopsy‐proven cases of GA by searching the pathology lab file of Shahid Faghihi hospital in Shiraz, Southern Iran, since December 2010 to March 2020. We recorded location of the lesions, demographic features including age, sex, and the clinical differential diagnosis. The pathology slides were reviewed once more with two expert dermatopathologists to determine the subtype of GA and their histologic features.

Inclusion criteria was biopsy‐proven GA, and exclusion criteria consisted of unavailable or poor quality pathological slides or specimen that makes the review impossible.

### 3.2 Data collection

All data regarding patients' sex, age, differential diagnosis, biopsy site, and their histologic features were recorded using SPSS version 26. No and 1+ mucin, eosinophils, and plasma cells were considered as not significant while 2+ and 3+ mucin, eosinophils, and plasma cells were considered as significant. Minimal and mild inflammation were considered as non‐significant, and moderate and severe inflammation were considered as significant inflammation. Detection of no, rare, and few giant cells were considered as non‐significant, and detection of some and many giant cells were considered as significant.

### 3.3 Statistical analysis

All data were analyzed using the chi‐square test. Descriptive statistics were reported as frequency (percentage) and analytical statistics were reported as mean ± standard deviation. A two‐sided *p*‐value of less than 0.05 was considered statistically significant.

## 4 RESULTS

One hundred‐forty‐nine patients were enroled. Slides of 13 cases could not be retrieved and therefore were excluded from the study. Thus, a total number of 136 patients were evaluated. Female/male ratio was 3.85. Interstitial GA was detected in 88 (64.7%) cases and 48 (35.3%) specimen showed features of complete GA. Clinical and histological characteristics of GA cases are presented in Table 1.

**TABLE 1:** Clinical and histological characteristics of granuloma annulare patients and specimens.

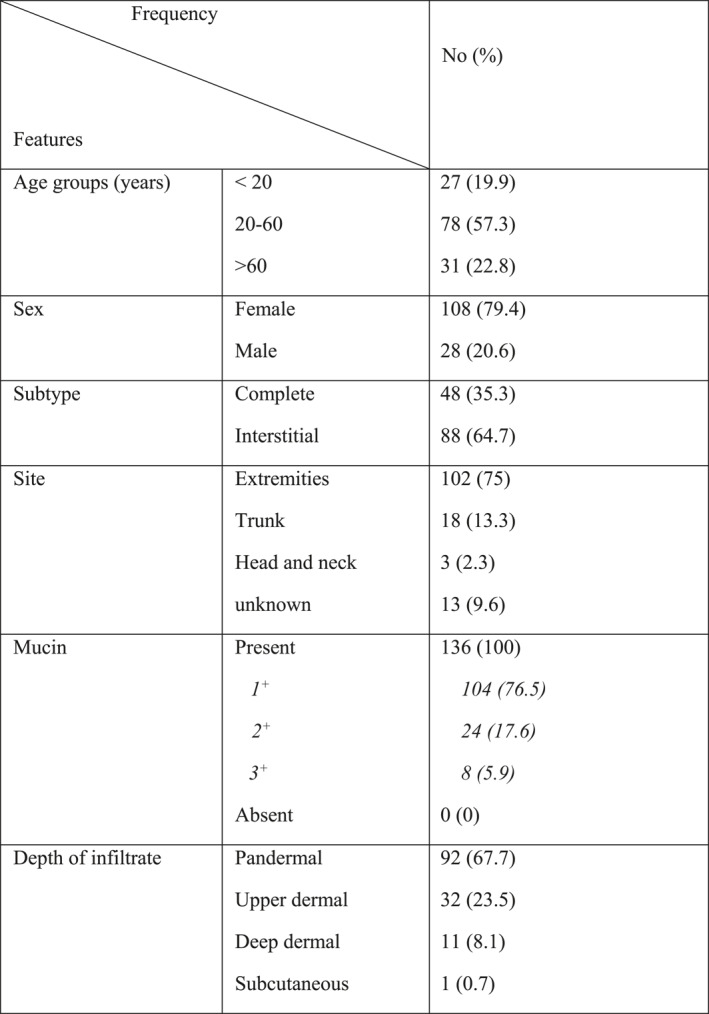 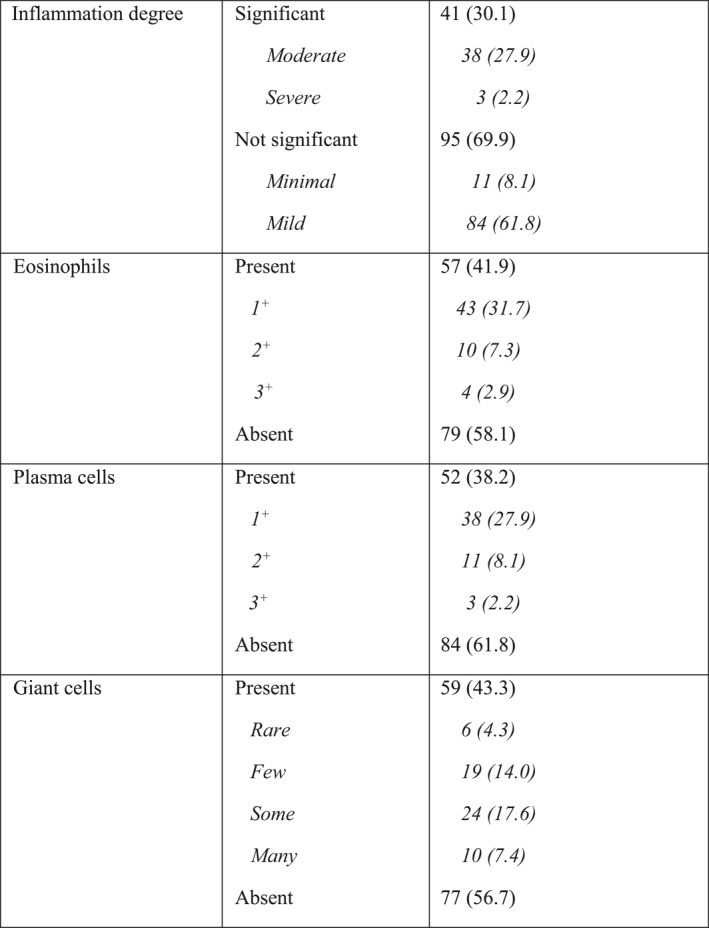

The most common clinical differential diagnoses were GA (68.4%), morphea (19.1%), eczema (18.4%), erythema annulare centrifugum (13.2%), and sarcoidosis (10.3%).

Seventy‐one (65.7%) female patients and 18 (64.3%) male patients had interstitial G.A; while 38 (35.2%) female patients and 10 (35.7%) male patients had complete GA.

Mucin was detected in 100% of patients. As Table 2 shows, significant mucin was detected more commonly in complete GA (33.3%) compared to interstitial GA (15.9%) (*p* = 0.019).

**TABLE 2:** Comparison of different histological features between complete and interstitial type of granuloma annulare.

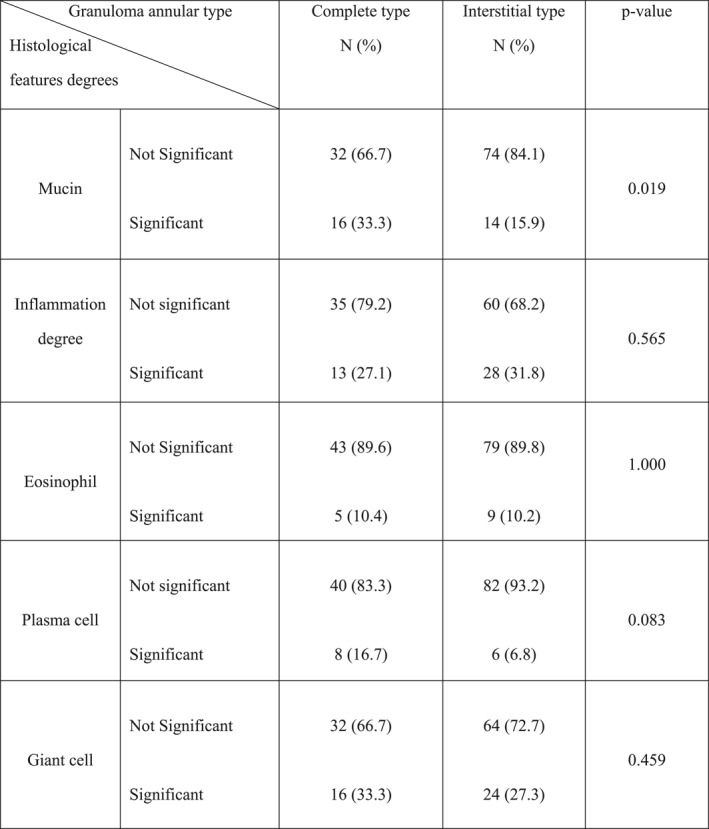

All patients (100%) had some degree of perivascular inflammation. There was no relation between inflammation degree, eosinophil, plasma cell, and giant cell in GA subtypes (*p*‐value >0.05).

The degree of inflammation, eosinophil density, and plasma cell density were not different in different genders (*p*‐value >0.05).

Significant degree of inflammation contained more significant plasma cells (*p*‐value = 0.006), but not more significant eosinophils (*p*‐value = 0.55) (Table 3).

**TABLE 3:** Relation between inflammation degree and degrees of eosinophils and plasma cells inflammation in granuloma annulare specimens.

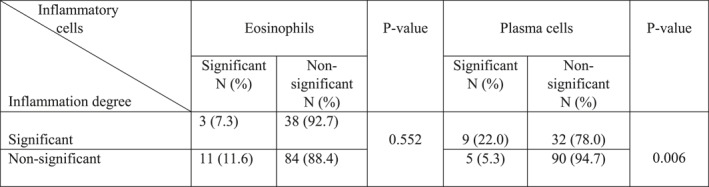

More dense plasma cells were related with milder degrees of eosinophil infiltration; however, we detected no significant relation between density of eosinophils and plasma cells (*p*‐value = 0.848).

Mucin density, inflammation degree, eosinophil, and plasma cell density were not significantly different in different age groups (*p*‐value >0.05). Significant plasma cells were mostly seen in the patients older than 60 years of age (50%). However, this difference was not significant (*p*‐value = 0.051). The most significant giant cells (52.5%) were seen in the 20–60 years age group (*p*‐value = 0.015) (Table 4).

**TABLE 4:** Comparison of different histological features in granuloma annulare age group.

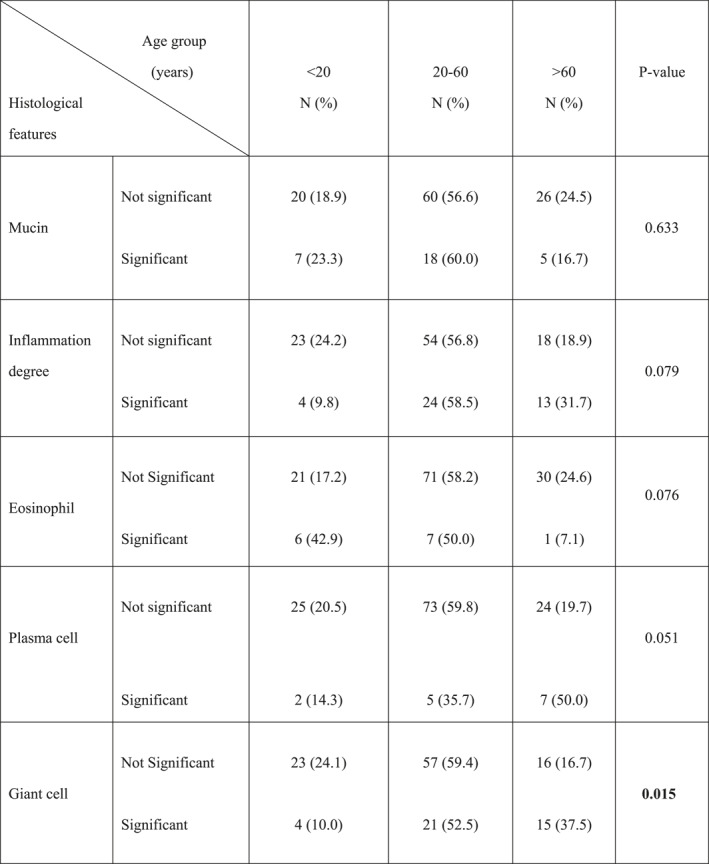

Three cases had neutrophils (± nuclear debris) in the necrobiotic areas (2.2%). Two cases had signs of focal leukocytoclastic vasculitis and deep dermal necrotizing vasculitis (1.5%). Four cases had foci of sarcoidal granuloma (2.9%).

Extremities were the site of predilection in our patients. The most common location of the lesion in women was hand followed by dorsum of feet. While the most common location of the lesion in men was the dorsum of feet followed by hand. These relations were not statistically significant (*p*‐value = 0.63).

## 5 DISCUSSION

Granuloma annulare is a benign, relatively common skin eruption. The clinical subtypes are localized, generalized, subcutaneous, and perforating GA. Although GA can be diagnosed clinically in most of the cases, skin biopsy is used to confirm the diagnosis or in the lesions with atypical presentations. The biopsy can reveal palisading granulomas, collagen degeneration, mucin, and a lymphohistiocytic infiltrate.^9^, ^13^, ^14^

This study determined the clinical and histopathologic features in patients with GA for the first time in Iran. According to our study this disease predominantly affected middle‐aged persons. GA was more frequent in female compared to male patients and was more seen in extremities in both gender. Interstitial GA was the more common histological subtype, but significant amount of mucin was mostly detected in complete subtype of GA. More severe inflammation contained more plasma cells, and more dense giant cells were seen in middle aged patients. In study of Barbieri et al. the disease was more frequent in fifth decade of life and female was more affected than male (3:1)^15^; Most prevalent presentation in the 5th and 6th decades of life was also reported in the other studies.^2^, ^16^ These findings are similar to our research's findings. Female predominance is reported in several studies^2^, ^16^, ^17^ that is in concordance with our study. In line with our research, in other studies extremities were the most affected area.^16^, ^18^

Malignancy‐associated and childhood GA also presented on the extremities in most patients.^18^, ^19^

In most of the studies that assessed the histopathologic findings of GA, interstitial pattern was the more common type of GA and 33%–71% of cases demonstrated this pattern.^11^, ^16^, ^17^, ^19^, ^20^

However, Cheng et al.^21^ showed palisading histiocytes as a predominant type of infiltration in GA (63.6%). Also, necrobiotic GA was slightly more than the interstitial and mixed types in the GA cases not associated with malignancy.^18^ Mucin was observed in all of our cases in haematoxylin and eosin (H&E) stain; however, in previous studies it was reported in 61%–93%.^16^, ^19^, ^20^, ^21^ The difference between studies may be the result of detection of mucin in the H&E or mucin special stains. Mucin was detected more frequently in the complete type of GA, compared to the interstitial GA, both in our and previous studies.^11^, ^17^ Ronen et al.^17^ reported scant to absent mucin in the interstitial GA. Despite the more frequent mucin in the complete type, the difference with interstitial type was not significant in our study. Detection of mucin is considered a clue to differentiate GA from other dermatoses with palisading granuloma.^21^ Mucin was more frequently detected in the Malignancy‐associated GA compared to control group.^18^

In our study, 41.9% of patients had eosinophil that is higher than what other researchers observed in their studies (3.7%–38%).^16^, ^18^, ^19^, ^20^, ^21^ The possibility of insect bite or drug reactions are proposed as the causes of eosinophil‐rich GA (20). Giant cells (43.3%) were less frequent than some of the studies (57%–90%),^16^, ^18^, ^19^, ^20^ but higher than some other studies (15.9%).^21^ We detected plasma cells in 38.2% of our patients. Plasma cells are reported in few previous studies in 33% of specimen.^16^ Significant inflammation (30% of our specimen) was less than the similar studies (60%–70%).^16^, ^18^ Different definition and lack of objective tools to measure the inflammation degree may explain this disconcordance. Neutrophils were more frequent in some studies^20^ (33%), compared to our results. Umbert et al.^11^ observed polymorphonuclear leukocytes in the necrotic areas, but they were absent in infiltrations. Vasculitis was a prominent feature in some studies (10%–20%),^16^, ^20^ but we detected few cases of vasculitis in our GA specimens.

The depth of infiltration is presented in different studies. Chaitra et al.^20^ reported the upper and mid‐dermal infiltrate in 53% of cases. Another study showed 60% upper and mid‐dermal, 10% upper dermal, and 30% pandermal infiltrate.^16^ The infiltration was reported in different depths of dermis in childhood GA.^19^ The infiltrate in our cases was mostly pandermal, followed by upper dermal infiltrate.

In our study, there was no significant correlation between inflammation degree, eosinophil, plasma cell, giant cell and different subtypes of GA.

We note some limitations to our study. First, we didn't have access to the records of associated diseases of patients. Second, we couldn't review the follow‐up of patients to find out the result of their treatment.

In conclusion, in our study, the prevalence of GA in women was higher than in men, and mostly located on the extremities. Significant amount of mucin was in favour of complete type of GA. More severe inflammation contained more plasma cells, and more dense giant cells were seen in middle aged patients. The inflammation was less severe and the infiltrate was mostly pandermal in our GA cases.

## CONFLICT OF INTEREST STATEMENT

None to declare.

## AUTHOR CONTRIBUTIONS

**Fatemeh Sari Aslani**: Conceptualization (equal); data curation (equal); investigation (equal); project administration (equal); resources (equal); supervision (equal); validation (equal); visualization (equal). **Fatemeh Pouraminaee**: Data curation (equal); formal analysis (equal); investigation (equal); software (equal); writing – original draft (equal); writing – review & editing (equal). **Mozhdeh Sepaskhah**: Conceptualization (equal); data curation (equal); investigation (equal); project administration (equal); resources (equal); validation (equal); writing – review & editing (equal). **Sheida Khosravani Ardakani**: Writing – original draft (equal).

## ETHICS STATEMENT

The study was approved by the Institutional Review Board (IRB) of Shiraz University of Medical Sciences (IR.SUMS.MED.REC.1399.387).

## Data Availability

The data that support the findings of this study are available from the corresponding author upon reasonable request.
